# Effects of Temperature, Relative Humidity, and Carbon Dioxide Concentration on Growth and Glucosinolate Content of Kale Grown in a Plant Factory

**DOI:** 10.3390/foods10071524

**Published:** 2021-07-01

**Authors:** Milon Chowdhury, Shafik Kiraga, Md Nafiul Islam, Mohammod Ali, Md Nasim Reza, Wang-Hee Lee, Sun-Ok Chung

**Affiliations:** 1 Department of Agricultural Machinery Engineering, Graduate School, Chungnam National University, Daejeon 34134, Korea; chowdhurym90@cnu.ac.kr (M.C.); nafiulislam@cnu.ac.kr (M.N.I.); sdali77@o.cnu.ac.kr (M.A.); reza5575@cnu.ac.kr (M.N.R.); wanghee@cnu.ac.kr (W.-H.L.); 2 Department of Smart Agricultural Systems, Graduate School, Chungnam National University, Daejeon 34134, Korea; kiragashafik@o.cnu.ac.kr

**Keywords:** *Brassica*, plant growth, glucosinolates, protected horticulture, environmental conditions

## Abstract

The growth of plants and their glucosinolate content largely depend on the cultivation environment; however, there are limited reports on the optimization of ambient environmental factors for kale grown in plant factories. This study was conducted to investigate the effects of temperature, relative humidity, and the carbon dioxide (CO_2_) concentration on kale growth and glucosinolate content in different growth stages of cultivation in a plant factory. Kale was grown under different temperatures (14, 17, 20, 23, and 26 °C), relative humidities (45, 55, 65, 75, and 85%), and CO_2_ concentrations (400, 700, 1000, 1300, and 1600 ppm) in a plant factory. Two and four weeks after transplantation, leaf samples were collected to evaluate the physical growth and glucosinolate contents. The statistical significance of the treatment effects was determined by two-way analysis of variance, and Duncan’s multiple range test was used to compare the means. A correlation matrix was constructed to show possible linear trends among the dependent variables. The observed optimal temperature, relative humidity, and CO_2_ range for growth (20–23 °C, 85%, and 700–1000 ppm) and total glucosinolate content (14–17 °C, 55–75%, and 1300–1600 ppm) were different. Furthermore, the glucosinolate content in kale decreased with the increase of temperature and relative humidity levels, and increased with the increase of CO_2_ concentration. Most of the physical growth variables showed strong positive correlations with each other but negative correlations with glucosinolate components. The findings of this study could be used by growers to maintain optimum environmental conditions for the better growth and production of glucosinolate-rich kale leaves in protected cultivation facilities.

## 1. Introduction

**Figure 1:**
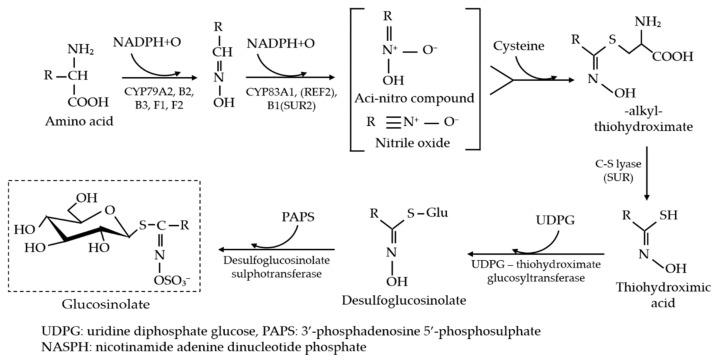
Synthesis of glucosinolates in Brassicaceous plants [11].

Kale (*Brassica oleracea* var. *alboglabra Bailey*) is a salad species that is one of the most versatile and commercially valuable vegetables due to its short growth period, various uses, and desirable metabolic and nutritional profiles [1,2,3]. This crisp and hearty vegetable is often consumed raw in salads and smoothies but can also be consumed in steamed, sautéed, or cooked states. Kale originates from China and has since gained particular attention in other countries due to its constituent cancer-preventive and human-health-promoting phytochemicals (i.e., glucosinolates, carotenoids, phenols, and vitamins) [4,5,6]. Glucosinolates are amino-acid-derived, active secondary metabolites that mainly contain sulfur- and nitrogen-related compounds (i.e., β-D-thioglucose, tryptophan, phenylalanine, sulfonated oxime moiety). They can be classified into aliphatic, aromatic, and indole groups [7], where each group consists of several chemical constituents. Progoitrin, sinigrin, glucoraphanin, and gluconapin are the major constituents of the aliphatic group. Similarly, 4-hydroxyglucobrassicin, glucobrassicin, 4-methoxyglucobrassicin and neoglucobrassicin, and gluconasturtiin are the major indole and aromatic glucosinolate constituents, respectively [1]. Glucosinolates are composed of relatively few amino acids and chain-elongated homologs through an independent metabolic pathway (Figure 1) and are available in all parts of almost all varieties of plants of the Brassicales order; however, the content is higher in the reproductive tissues (i.e., flowers and seeds) than in vegetative tissues [8]. The breakdown products of glucosinolates have a significant amount of anticarcinogenic activity for decreasing the risk of developing lung, stomach, colon, and rectum cancers; helping to maintain low blood pressure and reducing the risk of developing type 2 diabetes [6,9,10].

Kale growth and the formation of glucosinolates depend on crop genetic factors, tissue type, crop health, agronomic factors (i.e., water supply and fertigation), cultivation facilities (i.e., plant factory, greenhouse, and open field), and environmental factors such as temperature, relative humidity, carbon dioxide (CO_2_), light type, intensity, photoperiod, and cultivation methods [12,13,14]. The physical development stage is also a major determinant of the glucosinolates composition in kale [15]. Although kale can be easily cultivated in open fields using traditional methods, the quality and quantity of the growth and glucosinolate content cannot be ensured, as they are extremely sensitive to climatic and field conditions [16]. In recent years, farmers have produced kale in protected cultivation facilities, such as plant factories and greenhouses, due to the possibility of adjusting the growth environment and achieving fast and sustainable growth rates, functional component-rich and high-quality yield, lower rates of disease and pest infestation, and lower labor costs in addition to the possibility of year-round production with minimum influence from geological and climatic conditions [17,18,19]. Moreover, hydroponic cultivation systems with ion-specific (ISE-sensor-based) nutrient management could enhance the growth and nutritional profile of kale by 15% to 60% [20,21,22,23,24,25]. However, major environmental factors (i.e., temperature, relative humidity, and CO_2_) have to be specifically optimized according to crop to ensure sustainable kale growth and glucosinolate formation.

The physical growth of kale can be easily determined by measuring its physical properties such as plant length, width, weight, number of leaves, and stem diameter, whereas the glucosinolate content needs to be identified by laboratory analysis. The deposition of glucosinolates in growing plants and their distribution to plant organs are significantly affected by environmental factors [26], with temperature being one of the key factors. Several studies have been conducted to determine the process and effects of temperature on seed germination, physical development, flower formation, and yield [27,28,29,30]. However, physiological processes and their integration are sped up under higher temperatures with both positive and negative effects. For example, high temperatures promote faster growth and greater fruit production of plants, especially in cereal crops, but they also remove functional components from leaves through high transpiration rates [31]. Generally, elevated temperatures affect the structural components of chloroplasts significantly, causing effects such as variation in thylakoids, granum stacking, and swelling with photosystem II reduction, resulting in disruption to the cellular cytoplasm, cell breakdown and, ultimately, cell death. In addition, rising temperatures interrupt protein mechanisms, RNA synthesis, enzymatic interactions, and cell function. As a result, these imbalances and abnormal cell functioning affect the growth and accumulation of glucosinolate synthesis [32,33].

The relative humidity of the ambient environment also directly affects plant growth by resisting water and nutrient consumption. During transpiration, the relative humidity level becomes saturated. As a result, plants halt transpiration and nutrient uptake from the soil or growing media at high relative humidity levels where there is a lack of air circulation, resulting in gradual rotting in cases of long-term humidity saturation [34,35,36]. The maintenance of optimum relative humidity is essential for better growth and glucosinolate accumulation. Several researchers have reported that the photosynthesis rate is proportional to the relative humidity level as a higher range of relative humidity lowers water stress in the leaves and increases stomatal conductance. Although higher relative humidity increases the nutrient concentration, the nutrient solution supply and plant transpiration rate need to be monitored carefully [34,37].

The CO_2_ concentration influences the photosynthetic rate, metabolism, and physiological and chemical defense of plants [13,38]. A lack of CO_2_ would not only result in a lower biomass but the plants would also be of inferior quality and strength. As an essential substrate of the photosynthesis process, CO_2_ is directly absorbed by plants. CO_2_ also influences the transpiration process of plants. A meta-analysis was conducted, and it was reported that elevated CO_2_ could reduce transpiration by up to 22% in different plant species [39]. CO_2_ also preserves the essential nutrient components along with water by reducing the transpiration rate [40,41]. La et al. [38] investigated the effects of CO_2_ elevation at different nitrogen levels on the growth and glucosinolate content of Chinese kale and reported that all physical growth variables significantly increased with the elevation of CO_2_ at each nitrogen level; however, total glucosinolate content was only increased under low nitrogen level and elevated CO_2_ concentration.

The temperature, relative humidity, and CO_2_ concentration are the basic environmental factors that affect kale growth and, especially, glucosinolate formation. As they are interrelated, these factors should not be studied in isolation. The proper combination of these factors needs to be specifically confirmed for each crop to ensure optimal growth, a favorable nutritional profile, and identification of the ideal harvesting time. To date, very few studies have investigated the effects of these environmental factors on kale, especially when grown in plant factories using hydroponic cultivation methods. Therefore, the objective of this study was to investigate the effects of temperature, relative humidity, and CO_2_ on the growth and glucosinolate content at different stages of kale growth based on cultivation in a plant factory. 

## 2. Materials and Methods

### 2.1. Plant Factory and Seedling Preparation 

**Figure 2:**
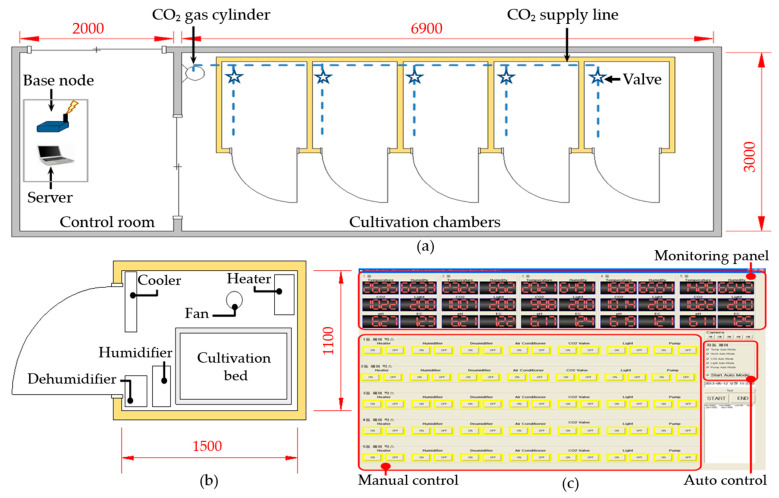
(**a**) Layout of the plant factory (control room and cultivation chambers); (**b**) fabricated individual chamber; and (**c**) ambient environment monitoring and control system. All dimensions are presented in millimeters (mm).

Plant factories are fully-closed crop cultivation systems that are fitted with artificial lights and used to grow high-value vegetables and medicinal plants throughout the year by utilizing artificially controlled ambient environmental factors [17,19]. In this study, five small chambers were prepared, as shown in Figure 2, to implement five different treatment conditions with varied temperature, relative humidity, and CO_2_ concentrations. The targeted environmental factors (i.e., temperature, relative humidity, and CO_2_) and other environmental factors (i.e., light sources, light intensity, photoperiod, and nutrient solution (EC and pH)) were maintained according to the experimental plan (Table 1). A wireless sensor network (XBee-Pro, Digi, Hopkins, MN, USA) was used to monitor the ambient environmental parameters and control the relevant actuators, as detailed by Chung et al. [42]. Three plant beds were placed vertically in each cultivation chamber, and a nutrient solution tank was kept at the bottom (floor). Each plant bed had 24 planting positions and 6 mist spray nozzles for spraying the nutrient solution onto plant roots as a fine mist for a duration of 2 min at 13-min intervals. Commercial nutrient solutions A and B (Daeyu Co., Ltd., Seoul, Korea) were used, and the target nutrient level was monitored and managed once a day using EC and pH sensors.

**Figure 3:**
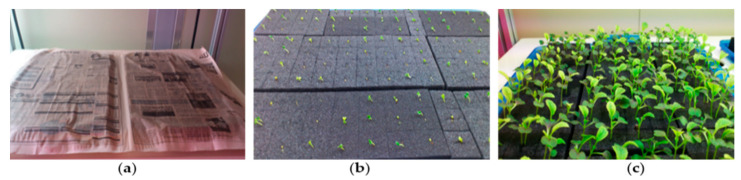
Preparation of kale seedlings for transplantation: (**a**) kale seeds were sown and covered; (**b**) germinated seeds; and (**c**) two-week-old seedlings under controlled environment conditions.

A commercial kale variety with green smooth leaves and a hard and fibered stem was cultivated in the experiments using a recycle-type aeroponic nutrient management system. Kale seeds were sown in a hydroponic germination sponge, covered with wet paper (until germination), kept in the plant factory under a controlled environment for germination, and grown until transplantation (Figure 3). The maintained temperature, relative humidity, CO_2_ concentration, light type, and photoperiod were 25 ± 3 °C, 65 ± 5%, 1000 ± 100 ppm, fluorescent, and 16/8 (day/night hours), respectively. Nutrient-rich water was provided into the root zone, and the EC and pH of the nutrient solution were 1.2 ± 1.00 (dS m^−1^) and 6.5 ± 0.5, respectively. After three weeks of germination, healthy seedlings with true leaves were transplanted into the plant bed with the sponge.

### 2.2. Experimental and Analytical Procedures 

#### 2.2.1. Experimental Design 

Different separate experiments were conducted to investigate the influences of temperature, relative humidity, and CO_2_ on kale growth and glucosinolate content. Five treatments with various environmental factors were applied in each experiment. For example, temperatures of 14, 17, 20, 23, and 26 °C were varied while other factors were kept constant. Similarly, five relative humidity levels and CO_2_ concentrations were implemented in experiments 2 and 3 to evaluate the effects of relative humidity and CO_2_, respectively. The targeted and monitored levels of temperature, relative humidity, and CO_2_ along with other growth factors are summarized in Table 1. The light source and ratio, intensity, photoperiod, and pH and EC levels were selected following the findings of Zhang et al. [43], Lefsrud et al. [44], Naznin et al. [45], and Jones [24], respectively. 

#### 2.2.2. Sample Collection and Data Acquisition

**Figure 4:**
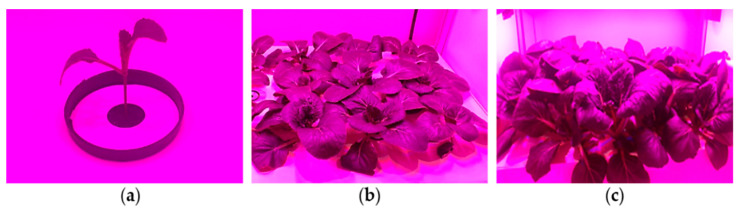
Growth condition of kale after different periods of transplantation: (**a**) transplantation day; (**b**) 2 weeks after transplantation; and (**c**) 4 weeks after transplantation.

Two and four weeks after transplantation (Figure 4), sample collection was performed in two steps. First, mature and healthy plants were visually selected and collected from the plant beds for physical growth evaluation. Three plants from each bed and nine plants from three beds (replicates) of each cultivation chamber were collected randomly among 72 plants (24 plants/bed × 3 plant beds). To analyze the glucosinolate content, three normal-sized, mature, healthy leaves were harvested from each plant bed (one leaf from each collected plant), and a total of nine leaves were collected from three plant beds (as a replication) from each cultivation chamber. The measured values for each growth parameter and the glucosinolate content were averaged to represent one data point. As a result, nine data points for growth parameters and one data point for the glucosinolate content were recorded from each plant bed. In total, 270 data points were collected for growth evaluation (9 data points/bed × 2 sampling times × 3 replications × 5 treatments) and 30 data points were collected to assess the glucosinolate content (1 data point/bed × 2 sampling times × 3 replications × 5 treatments) for each experiment. The physical growth variables, namely, the plant height, width, weight, number of leaves, stem diameter, chlorophyll level, leaf length, width, and weight were measured, and the leaves were transferred to the chemical laboratory immediately (to minimize the degradation) for glucosinolate analysis using a commercial high-performance liquid chromatography (HPLC) machine (model: 1200 series, Agilent Technologies, Santa Clara, CA, USA). The chlorophyll concentration was also measured using a commercial device (model: SPAD 502DL, Spectrum Technology Inc., Aurora, IL, USA). 

#### 2.2.3. Estimation of Glucosinolate Content

The glucosinolates of the freshly harvested kale leaves were extracted and analyzed as described by Doheny-Adams et al. [46]. The whole process was conducted according to the ISO 9167:2019 [47], and the process is divided into four major steps: (a) tissue disruption, (b) extraction in methanol, (c) purification and desulfation, and (d) separation and identification of glucosinolates by HPLC analysis (1200 series, Agilent Technologies, Santa Clara, CA, USA). The collected leaf samples were stored in an airtight box, taken to the chemical laboratory immediately to freeze in liquid nitrogen, and stored at −80 °C for 48 h to reduce the activity of myrosinase. For freeze-drying, samples were lightly wrapped with aluminum foil and transported on dry ice to load into the freeze drier (Lyotrap, LTE scientific Ltd., Oldham, UK) within 30 s. The freeze-dried leaf samples were ground to make a homogenized fine powder using a grinder (EK2311, Salter, Tonbridge, UK). Then, 100 mg of the freeze-dried samples was preheated for 3 min at 75 °C and 4.5 mL of preheated 70% methanol at 75 °C was added. The sample was incubated for 10 min at 75 °C (with manual shaking every 2 min) and then centrifuged by a rotor at 4000 rpm (B 3.11, Jouan, Nantes, France) for 10 min. In the purification step, 25 mg of sulfatase and 1ml of 40% ethanol were mixed and centrifuged for 1 min at 8000 rpm. The supernatant was shifted to a new Eppendorf tube and 1 mL of pure ethanol was injected for precipitating the sulfatase before the second centrifugation. Finally, the sulfatase pellet was air dried after separating from the supernatant and diluted in 2 mL of water. For desulfation, 0.5 cc of Sephadex slurry was used to prepare the columns and 2 mL of imizadole formate (6 M) was added on each for activation. The columns were cleaned twice with 1 mL of water each time. The columns were washed again using 1 mL of 20 mM sodium acetate, and 75 μL of purified sulfatase (0.05–0.3 U/mL) was injected. After that, columns were incubated for 24 h at 28 °C before desulfoglucosinolates were eluted with two 1 mL volumes of water. After 24 h of incubation, elution of desulfoglucosinolates was performed thrice using 1.5 mL of distilled water and filtered through 0.45-μm polytetrafluoroethylene (PTFE) syringe filters (Millipore, Bedford, MA, USA) into an HPLC vial. A reverse phase C18 column (150 × 3.0 mm, 3 µm, Inertsil ODS-3, GL Sciences, Tokyo, Japan) was used, which was equilibrated for 30 min using ultrapure water (solvent A) and 100% acetonitrile (solvent B) with detection at 227 nm. The flow rate was 0.4 mL min^−1^, and separation was performed according to the default program. As an external standard, sinigrin (0.1 mg/mL; Sigma, St. Louis, MO, USA) was utilized. The identification and quantification of individual glucosinolate components was performed by comparing the sinigrin retention time and using their HPLC areas and response factor, respectively. In this study, the obtained retention time for progoitrin, sinigrin, glucobrassicin, 4-methoxyglucobrassicin, and neoglucobrassicin were 5.97, 7.13, 21.93, 24.68, and 30.37 min, respectively.

#### 2.2.4. Statistical Analysis

All the presented physical growth parameters and glucosinolate content values are the means of independent measurements for different treatments of each environmental factor. The significance of differences between mean values was determined by two-way analysis of variance (ANOVA). Data were analyzed considering 95% confidence levels and two-sided confidence intervals. Duncan’s multiple range test was used to simultaneously compare means (SAS Institute Inc, Campus drive Cary, NC, USA). A correlation matrix recording correlation coefficients was created to show the inter-relationships between variables. 

## 3. Results

### 3.1. ANOVA of the Environmental Factors

The effects of ambient environmental factors (temperature, relative humidity, and CO_2_) on plant physical growth variables and total glucosinolate content were analyzed using two-way ANOVA analysis. Five different treatment conditions for each environmental factor and two sampling times were considered when conducting the ANOVA analysis for each growth variable and the glucosinolate content. The results of the two-way ANOVA analysis for the plant height, width, weight, and total glucosinolate content are shown in Table 2 out of nine physical variables and five identified glucosinolate components. The F-values of the treatments and sampling times were higher than the F crit values, except for some growth and glucosinolate variables under the CO_2_ treatments, which confirms the adequacy of the hypothesis. This ANOVA analysis indicates that the treatments and sampling times had significant impacts (*p* < 0.05) on the growth and glucosinolate content (except for some CO_2_ treatments). However, some P-values under the CO_2_ treatments were higher than 0.05, which also indicates that those growth or glucosinolate variables were not notably affected by the CO_2_ treatments. The overall results show that a single unit change of each environmental factor will affect the plant growth and glucosinolate content.

### 3.2. Correlation of the Glucosinolates Components

Table 3 shows the magnitude, direction, and linear pairwise relationship between the identified glucosinolate variables under the considered ambient environmental factors (temperature, relative humidity, and CO_2_). Among the five identified glucosinolate variables under the temperature experiments, sinigrin and glucobrassicin were strongly and positively correlated among them and identified glucosinolate variables under the relative humidity experiments, and they were strongly and negatively correlated with the variables identified in the CO_2_ experiments. The correlations were statistically significant at a 0.1% level (except for some variables). Although, progoitrin had a significant positive correlation with each of the five identified glucosinolate variables under the CO_2_ experiments, no significant correlations were observed with other variables. Except for some strong correlations, 4-methoxyglucobrassicin, and neoglucobrassicin were also not significantly correlated with other identified glucosinolate variables. Strong negative correlations with a 0.1% significance level were observed for most of the identified glucosinolate variables under the relative humidity and CO_2_ experiments. However, identified glucosinolate variables under the CO_2_ experiments were strongly and positively correlated. They were statistically significant at a 0.1% level (except for the C_Sin). The multicollinearity issue can also be predicted from the correlation matrix. A highly correlated value (>0.7) hinders the evaluation of the true effects of the predictor variables. According to Table 3, some of the glucosinolate variables had notable evidence of strong correlation. For example, C_Pro showed positive correlations of 0.90, 0.90, and 0.99 with C_Glu, C_4-met, and C_Neo, respectively, and C_4-met showed negative correlations of −0.91 and −0.93, with T_Sin and T_Glu, respectively. Variance inflation factor (VIF) was also investigated and the values varied from 1 to 3 for most of the variables, indicating that the variables were slightly explained by other independent variables. However, the VIF values of T_Sin (4.72) and T_Glu (6.46) were relatively high [48].

### 3.3. Evaluation of Temperature Effects

A statistical analysis was conducted to evaluate the effects of temperature on kale growth, and the results are shown in Table 4. Regarding kale physical properties, an overall high growth rate was observed at 20–23 °C, and the lowest growth rate occurred at 14 °C. However, some physical parameters showed greater numbers at 17 and 26 °C. They were plant height (17 °C) and width (26 °C) after two weeks of transplantation, and chlorophyll level (17 °C), leaf length and weight (26 °C) after four weeks of transplantation. The data points of no. of leaves, stem diameter, and leaf parameters (length, width, and weight) were very close to the mean (low standard deviation); however, the data points of other growth variables, especially plant height and width, were spread out over a wide range of values. Standard deviation was greater in samples collected after four weeks of cultivation, compared to the two weeks. According to Duncan’s range test results, significant differences were observed for the plant width, weight, and leaf parameters (length, width, and weight) at 2-week sampling time, and the plant height, and leaf parameters at 4-week sampling time, depending on the temperature levels. Contrariwise, the rest of the growth variables (specifically the number of leaves, stem diameter, and chlorophyll level) did not show statistical significance regarding the temperature variations.

**Figure 5:**
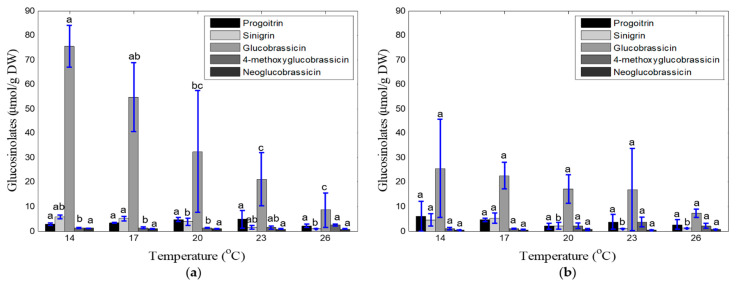
Concentrations of glucosinolate components (μmol/g DW) under different temperature treatments and cultivation periods: two weeks after transplantation (**a**) and four weeks after transplantation (**b**). ^a, b, c^ levels of components with the same letters are not significantly different at *p* < 0.05.

Figure 5 shows the effects of temperature on the glucosinolate content, based on various components, of harvested kale leaves after two and four weeks of transplantation. Glucobrassicin was found to be a dominant glucosinolate component in both cases. However, an inverse relationship was observed between the contents of all glucosinolate components and increased ambient temperature levels and cultivation period. The total glucosinolate content became lower at each increased temperature level, and the lowest total glucosinolate of kale leaves in each sampling time was observed at 26 °C. A high standard deviation trend was observed for each glucosinolate component due to the low sampling number. Among the five levels of temperature, the total glucosinolate content was higher at 14–17 °C in both cultivation periods. According to Duncan’s range test, the concentrations of sinigrin, glucobrassicin, and 4-methoxyglucobrassicin were significantly different for each temperature level in samples collected after two weeks of cultivation; however, no significant differences were observed among the other glucosinolate components (except sinigrin at 4th week) for different temperature levels and cultivation periods.

The interactions of each growth variable and glucosinolate component under the different temperature treatments after four weeks of transplantation were analyzed using the correlation matrix and the results are summarized in Table 5, where the level of significance and VIF are also mentioned. In many cases, strong positive and negative correlation coefficients were observed. The physical growth variables were found to be strongly correlated with each other (around 0.50–0.98). However, the chlorophyll level showed negative correlations with all the growth variables, and positive correlations were observed with progoitrin, sinigrin, and glucobrassicin. The progoitrin, sinigrin, and glucobrassicin were strongly positively correlated with each other and negatively correlated with 4-methoxyglucobrassicin and neoglucobrassicin. Moreover, the detected glucosinolate components (except 4-methoxyglucobrassicin) were negatively correlated with most of the physical growth variables. According to the VIF analysis, most of the variables were moderately correlated; however, some variables (i.e., leaf length and glucobrassicin) were highly correlated, which might adversely affect other variables.

### 3.4. Evaluation of Relative Humidity Effects

The effects of relative humidity on kale growth properties are summarized in Table 6. The growth status was evaluated at two different stages (two and four weeks after transplantation). Most of the physical growth variables were prominent at the 85% relative humidity level at both sampling periods, except for the number of leaves, stem diameter, chlorophyll level (55%), and leaf length (75%) in the second week, and the number of leaves, leaf weight (65%), and chlorophyll level (45%) in the fourth week of cultivation. Except for some growth variables, the overall lowest growth performance was observed at the 45% relative humidity level in both sampling times. The data points of some growth variables, such as plant height and width, leaf length and width, were spread out over a wide range compared to other growth variables, and greater standard deviations were observed in samples collected after four weeks of cultivation, compared to the two weeks of cultivation. According to Duncan’s range test, all the growth variables (except the chlorophyll level) were significantly different at the 2-week sampling time depending on the relative humidity levels. A similar result was observed (except for the plant weight, chlorophyll level, and leaf width) at the 4-week sampling time.

**Figure 6:**
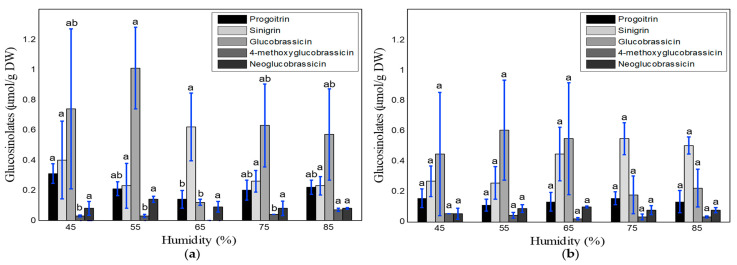
Concentrations of glucosinolate components (μmol/g DW) under different relative humidity treatments and cultivation periods: two weeks after transplantation (**a**) and four weeks after transplantation (**b**). ^a, b^ levels of components with the same letters are not significantly different at *p* < 0.05.

The results of the glucosinolate analysis for different relative humidity treatments and cultivation periods are shown in Figure 6. The aliphatic glucosinolates (i.e., progoitrin, sinigrin) and indole glucosinolate (i.e., glucobrassicin) were the most prominent components at both of the sampling times. The overall glucosinolate concentrations decreased slightly in the samples collected after the fourth week of cultivation. A high standard deviation was observed, especially for the glucobrassicin, as the sample number was low and sometimes all glucosinolate components were not detected in some samples. According to Duncan’s range test, no significant differences were observed among the glucosinolate components (except for the progoitrin, glucobrassicin, and 4-methoxyglucobrassicin at the 2-week sampling time) for different relative humidity treatments and cultivation periods. 

Table 7 shows the correlation matrix of physical and glucosinolate properties for different relative humidity treatments after four weeks of cultivation. All physical growth variables, except for the number of leaves and the chlorophyll level, showed strong positive correlations with one another. A fairly good correlation (both positive and negative) was observed between the physical variables and glucosinolate components. However, most of the glucosinolate components were negatively correlated with each other. Moreover, the VIF values were also determined. Except for the leaf length (VIF: 11.93), other predictors were moderately correlated, which resulted in a low influence on other independent variables.

### 3.5. Evaluation of CO_2_ Effects

A summary of the effects of CO_2_ treatments and cultivation periods on kale growth is given in Table 8. The overall growth performance was higher under 700–1000 ppm CO_2_. However, a notable growth rate of some parameters (i.e., chlorophyll level and leaf length at the 2-week sampling time, and plant height and chlorophyll level at the 4-week sampling time) was observed under 400 ppm of CO2. Relatively low growth performance was observed under 1300 and 1600 ppm of CO_2_ in both sampling periods. Besides this, the spread of standard deviations of the growth variables was almost similar for both of the sampling periods. Comparatively high standard deviations were observed for the plant parameters (height, width, and weight) and chlorophyll level compared to other growth variables. Based on Duncan’s range test results, there were no significant differences for the growth parameters (except the plant height at the 4-week sampling time) under different CO_2_ concentrations and cultivation periods.

**Figure 7:**
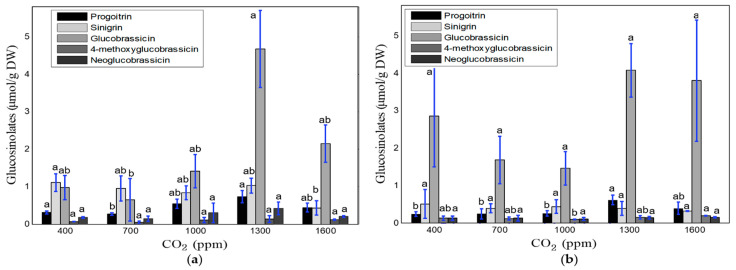
Concentrations of glucosinolate components (μmol/g DW) under different CO_2_ treatments and cultivation periods: two weeks after transplantation (**a**) and four weeks after transplantation (**b**). ^a, b^ levels of components with the same letters are not significantly different at *p* < 0.05.

The effects of different CO_2_ concentrations on the glucosinolate content are shown in Figure 7. The optimal CO_2_ level in relation to the total glucosinolate content was 1300 ppm at both the second and fourth weeks of cultivation. The progoitrin, sinigrin, and neoglucobrassicin contents decreased after the two weeks of cultivation. The low sampling number caused high standard deviations of the detected glucosinolate components. Glucobrassicin was found to be a dominant component in the samples collected after 4-weeks. The results of Duncan’s range test showed a significant difference in glucosinolate components (except for the 4-methoxyglucobrassicin and neoglucobrassicin at the 2-week sampling, and sinigrin, glucobrassicin, and neoglucobrassicin at the 4-week sampling time) under different CO_2_ concentrations and both cultivation periods.

Table 9 shows the interactions of each physical and functional parameter with one another, along with the significance levels for different CO_2_ treatments after four weeks of transplantation. Weak correlations (both positive and negative) were detected among most of the physical growth variables. However, the glucosinolate components (except sinigrin) were strongly positively correlated with each other and mostly negatively correlated with physical growth variables. According to the VIF analysis, the VIF values of the physical variables varied from 1 to 2, except plant height (4.23), indicating low correlations. Contrariwise, the VIF values of the glucosinolate components (except progoitrin) were comparatively high, which indicated highly correlated relationships and influences on other predictors.

## 4. Discussion

There is an interaction between plant growth and glucosinolate concentration, which strongly depends on the environmental conditions and water–nutrient consumption rate, along with the plant species, growth method, cultivation period, and cultivation facilities used [12,13,14,16,49,50]. In this study, the growth rate of kale increased with the cultivation period. The overall maximum growth rate was observed at 20–23 °C, around 85% relative humidity, and 700–1000 ppm CO_2_ (Table 4, Table 6 and Table 8). The optimal temperature, relative humidity, and CO_2_ range for total glucosinolate content were 14–17 °C, 55–75%, and 1300–1600 ppm. However, the glucosinolate content of kale decreased notably as cultivation period, temperature, and relative humidity level increased (Figure 5 and Figure 6). Contrariwise, it increased with increased CO_2_ concentration (Figure 7). All biological processes of plants speed up at higher temperatures [51]. However, the sensitivity of plants to the atmospheric temperature depends on the growth stage. Plants always seek to maintain a balance between the plant-body temperature and air temperature. If the plant is heated up, the transpiration rate increases to cool down plants, which increases water and nutrient uptake, resulting in phonological changes in plants [52,53]. This assimilation process occurs quickly in the early growth stage. We observed a high growth rate at 23–26 °C in the 2nd week, which was reduced along with the temperature range (to 20–23 °C) in the 4th week. However, the rapid transpiration process also ejects many nutrient components, which lowers the concentration of glucosinolate components, as shown in Figure 5 [33]. Conversely, a high level of accumulation of functional components (i.e., glucosinolates) occurs at low temperatures. Steindal et al. [29] explained that low temperatures activate cold acclimatization processes, including many biochemical and physiological changes, to improve the cold tolerance capacity. These procedures reduce the growth and accumulation of osmolytes and the functional component composition. In this study, the lowest rate of physical growth and the highest concentration of glucosinolates were also observed at 14 °C (Table 4, Figure 5). Velasco et al. [49] reported an inverse relationship between low temperatures and the total glucosinolate content. Relative humidity is directly related to CO_2_ acclimation through the stomata response, which is connected with plant growth and nutritional levels. Ahmed et al. [54] reviewed several studies and reported that a relative humidity of lower than 40% and higher than 85% causes stomatal malfunctioning, inhibiting the plant growth rate and photosynthesis. They also mentioned that the optimal range of relative humidity for leafy vegetables (i.e., lettuce) is 70–80%. In this study, maximal growth was found at a relative humidity range of 75–85%, and no significant difference was observed at a relative humidity range of 45–85% for the glucosinolate components (Figure 6), which matches the findings of previous studies. In addition to the effects of temperature and relative humidity, a significant impact of the CO_2_ concentration was observed on the accumulation of glucosinolate components rather than the growth rate of kale. In open environments, the concentration of CO_2_ remains constant (300–400 ppm), but this concentration can be increased in protected cultivation facilities (i.e., greenhouses and plant factories). Usually, the demand for CO_2_ increases with the increment of plant growth parameters and biomass [55]. In this study, CO_2_ concentrations of 700 to 1000 ppm were associated with better growth performance (Table 8), and higher glucosinolate formation was observed under 1300 to 1600 ppm range of CO_2_ (Figure 7). Higher concentrations of CO_2_ help to synthesize larger amounts of carbohydrates and other functional components through photosynthesis [13,38,40]. Moreover, lower reduction of the photosynthetic ingredients under elevated CO_2_ concentrations improves the glucosinolate content [39,41]. An overaccumulation of glucosinolates was observed under experiment-1 (temperature) compared to experiments-2 and 3 (relative humidity and CO_2_). We know glucosinolates are significantly affected by the variety, genetics, plant growth stage, irrigation level, growing media, and environmental variables (i.e., temperature, humidity, CO_2_, and light conditions). For example, Chen et al. [56] investigated the variation of glucosinolates in Chinese Brassica campestris vegetables (Chinese cabbage, purple cai-tai, choysum, pakchoi, and turnip) and reported that total glucosinolates varied from 14–130 mg/100 g fresh weight (FW), where He et al. [57] observed the minimum (28.9 µmol/100 g FW) in broccoli and maximum (278 µmol/100 g FW) in Chinese kale. From seedling to early flowering, the total glucosinolate content increased with plant age in *B. oleracea* leaves. After that point, the aliphatic glucosinolate content decreased dramatically over time as the glucosinolates transferred in the flower buds [49]. Qian et al. [58] investigated the effect of light quality on glucosinolate composition and content of Chinese kale sprouts under 23 °C temperature, 80% relative humidity, 16/8 h photoperiod, and red: blue: white light condition, and observed 167.32–288.70 and 72.66–87.48 μmol/g DW of total glucosinolates in shoots and roots, respectively. Similarly, temperature, humidity, and CO_2_ have an individual effect on glucosinolate components and accumulation. Rosa and Rodrigues [59] reported that the amount of glucosinolates increases 4–35% in the Brassica species in summer compared to winter seasons. They also observed 386 ± 71 μmol/100 g DW of total glucosinolates in the Chinese cabbage leaves under 20 °C, which increased up to 409 ± 104 μmol/100 g DW under 30 °C. The possible reason behind this increment is the proportional relationship between temperature and the photosynthesis rate. However, glucosinolate components and contents are degraded under both very hot and cold temperatures. Although the light types, intensity, photoperiod, and EC-pH were kept constant in this study (for experiments 1, 2, and 3), the variation of glucosinolate levels was observed due to the individual effect of temperature, humidity, and CO_2_. As the experiment 1, 2, and 3 were conducted separately, the overaccumulation of glucosinolates under temperature treatments might have occurred due to the overall growing condition; however, it is very important to maintain consistency between experiments. To minimize the inconsistency between experiments, the following measurements could be considered to handle and minimize the variations. First, similar seedlings could be prepared as much as possible, so that pre-transplanting cannot affect the final harvested product. Moreover, the number of samples could be increased by cultivating kale in bigger and multiple plant beds. Finally, maintenance of the same cultivation condition through more accurate and precise control of the environmental variables is necessary.

In the correlation matrixes, strong positive correlations were observed among all the physical growth variables, except for the chlorophyll level. Negative correlations with other growth variables were shown. The most likely reason for this phenomenon is that chlorophyll is an indicator of the health of the photosynthetic apparatus, and the concentration (amount per mass) is a function of the leaf area. As the midrib and petiole of kale (depending on the cultivar) are large, the midrib might become enlarged, diluting the concentration of chlorophyll in the lamina during the growth period, which results in negative correlations with other growth variables [60]. Moreover, the efficiency of chlorophyll varies over time due to the engagement–disengagement of assorted photoprotective mechanisms under fluctuating light conditions. This results in energy loss (absorbed by chlorophyll as heat) and affects carbohydrate (glucose) accumulation. This might be another reason for the negative correlation with kale growth [61,62]. Besides this, glucosinolate components were strongly positively correlated with each other under elevated CO_2_ concentrations, because glucosinolate synthesis is proportionally related to photosynthesis [38]. The mechanisms of biochemical reactions are, in fact, very complex, and in many processes, the biochemical pathways are only hypothetical or assumed, and the intermediate reactions and products are not fully known. At any stage in the biochemical chain, double bonds, which are very reactive, may be affected by temperature, relative humidity, and CO_2_ as well as by free radicals in the environment. Particularly, glucosinolate synthesis can be illustrated according to the following steps: (1) radical substitution and the addition of water occurs at elevated temperatures and/or in the presence of radicals; (2) addition reactions to carbon–nitrogen double bonds, resulting in carbonic acid esters; (3) electrophilic addition of water to double bonds that creates two new sigma bonds, resulting in the formation of alcohol; and (4) the occurrence of rearrangement, transposition, and isomerization involving double bonds, allyl radicals, and the glucose cycle. The increased reactivity of double bonds makes them very susceptible to environmental factors, specifically temperature, relative humidity, and CO_2_.

Cartea and Velasco [6] reported that the concentrations of glucosinolate components vary depending on genetics and environmental factors, along with the crop cultivation methods, harvest, storage, and even the processes of meal preparation. Velasco et al. [49] specifically showed that the concentrations of aliphatic glucosinolate components gradually increase in vegetative tissues (i.e., leaves) and are transferred to the reproductive tissues (i.e., flowers and seeds) during the flowering period. In addition, the indole glucosinolate components of leaves and flower buds gradually decrease after a certain period of cultivation. However, the concentrations of aromatic glucosinolates do not vary significantly with the cultivation period. In this study, kale was cultivated in the plant factory using an aeroponic method (one type of hydroponics). The fast growth rate due to proper ambient environment and nutrient management might be a reason for high glucosinolate accumulation in the early stage (two weeks after transplantation), and it gradually declined with the cultivation period (four weeks after transplantation). Determination of the proper harvesting time of brassicaceous plants has been investigated in several studies [63,64]. Based on the environmental factors and cultivation methods used in this study, early harvesting (2–3 weeks after transplantation) is suggested as a possible strategy to achieve glucosinolate-rich kale.

## 5. Conclusions

This study was conducted to investigate the effects of temperature, relative humidity, and CO_2_ on the growth and glucosinolate content of kale plants hydroponically grown in a plant factory, where five different treatments of each environmental variable were applied separately, and samples were collected after two different periods of cultivation. According to the results, the optimal temperature, relative humidity, and CO_2_ range for growth and total glucosinolate content were 20–23 °C, 85%, and 700–1000 ppm, and 14–17 °C, 55–75%, and 1300–1600 ppm, respectively. The glucosinolate content of kale was high in the early growth stage, with low temperature and humidity levels, and elevated CO_2_ concentrations. Strong positive correlations were observed among the physical growth variables, and weak correlations were found between the growth and glucosinolate parameters, which indicated that high physical growth might not ensure the high concentration of glucosinolates. According to the findings of this study, early harvesting (i.e., after 2 weeks of transplantation) could be preferred. As the optimum level of temperature, humidity, and CO_2_ was different in two- and four-week sampling times, dynamic ambient environment management might be adopted. Farmers could maintain the optimum range of each environmental variable separately based on their target (growth or glucosinolate level), or preferred combined management of the temperature, relative humidity, and CO_2_ during kale cultivation within protected cultivation facilities.

## Figures and Tables

**Table 1:** Different treatments of temperature, relative humidity, and CO_2_ during the kale cultivation in the plant factory.

Environmental Variables	Targeted Levels			Monitored Levels	Used Sensor
	Experiment 1 (Temp.)	Experiment 2 (Humi.)	Experiment 3 (CO_2_)		
Temperature (°C)	14 ± 1	20 ± 1	20 ± 1	14.58 ± 0.74	ETH-01DV, ECONARAE, Seoul, Korea
	17 ± 1			17.34 ± 1.80	
	20 ± 1			20.25 ± 0.69	
	23 ± 1			23.26 ± 0.52	
	26 ± 1			25.97 ± 1.64	
Relative humidity (%)	65 ± 5	45 ± 5	65 ± 5	44.78 ± 5.23	ETH-01DV, ECONARAE, Seoul, Korea
		55 ± 5		56.06 ± 4.35	
		65 ± 5		67.66 ± 4.67	
		75 ± 5		76.85 ± 4.49	
		85 ± 5		82.66 ± 5.65	
CO_2_ (ppm)	1000 ± 100	1000 ± 100	400 ± 100	475.62 ± 106.30	SH-300-DS, SOHA TECH CO. Ltd., Seoul, Korea
			700 ± 100	723.29 ± 140.60	
			1000 ± 100	980.75 ± 125.36	
			1300 ± 100	1318.34 ± 125.11	
			1600 ± 100	1672.30 ± 93.21	
Light source (LED color ratio)	R:B = 11:7			-	-
Light intensity (μmol m^−2^ s^−1^)	160			160 ± 25	GY-30, ROHM Co. Ltd., Kyoto, Japan
Photoperiod (day/night hrs)	16/8			-	MaxiRex 5QT, Legrand Korea Co., Ltd., Seoul, Korea
pH	6.50 ± 0.5			6.55 ± 0.52	PH-BTA, Vernier, OR, USA
EC (dS m^−1^)	1.2 ± 1.00			1.28 ± 0.29	CON-BTA, Vernier, OR, USA

**Table 2:** Two-way ANOVA test showing the individual effects of the treatments (Tr) and sampling times (ST) on growth variables and total glucosinolate content of kale.

SV	Plant Height			Plant Width			Plant Weight			Total Glucosinolates		
	Tr	ST	Err	Tr	ST	Err	Tr	ST	Err	Tr	ST	Err
**Temperature effect**												
**SS**	4.1 × 10^4^	2.3 × 10^5^	6.6 × 10^3^	1.4 × 10^4^	4.3 × 10^4^	3.2 × 10^3^	206.9	1.03 × 10^3^	298.2	8.7 × 10^3^	3.7 × 10^3^	3.2 × 10^3^
**df**	4	1	20	4	1	20	4	1	20	4	1	20
**MS**	1.0 × 10^4^	2.3 × 10^5^	334.1	3.5 × 10^3^	4.3 × 10^4^	162.1	51.71	1.3 × 10^3^	14.91	2.1 × 10^3^	3.7 × 10^3^	162.6
**F-value**	30.89	714.63		21.71	269.3		3.46	69.01		13.45	22.99	
***p*-value**	<0.001	<0.001		<0.001	<0.001		<0.05	<0.001		<0.001	<0.001	
**F crit**	2.87	4.35		2.87	4.35		2.87	4.35		2.87	4.35	
**Relative humidity effect**												
**SS**	5.1 × 10^3^	2.03 × 10^4^	4708	1.8 × 10^4^	1.1 × 10^5^	1.3 × 10^4^	6.53	192.53	7.33	0.34	1.16	1.93
**df**	4	1	20	4	1	4	4	1	20	4	1	20
**MS**	1.3 × 10^3^	2.03 × 10^4^	235.4	4607.4	1.1 × 10^5^	632.4	1.63	192.53	0.37	0.08	1.16	0.09
**F-value**	5.49	86.37		7.29	189.27		4.45	525.09		0.88	12.01	
***p*-value**	<0.05	<0.001		<0.001	<0.001		<0.05	<0.001		0.49	<0.05	
**F crit**	2.867	4.35		2.87	4.35		2.87	4.35		2.87	0.41	
**CO_2_ effect**												
**SS**	652.8	3020	1187.3	311.67	4.4 × 10^4^	1.4 × 10^4^	0.252	898.7	4.08	64.46	3.18	55.65
**df**	4	1	20	4	1	20	4	1	20	4	1	20
**MS**	163.2	3020	59.37	77.91	4.4 × 10^4^	748.5	0.06	898.7	0.204	16.11	3.18	2.78
**F-value**	2.75	50.8		0.10	59.31		0.31	4398.3		5.79	1.14	
***p*-value**	0.05	<0.001		0.97	<0.001		0.86	<0.001		<0.05	0.29	
**F crit**	2.87	4.35		2.87	4.35		2.87	4.35		2.87	4.35	

SV: source of variation, SS: sum of square, df: degree of freedom, MS: mean square, F crit: critical value in the F distribution, Tr: treatment, ST: sampling times, Err: error, E: exponential.

**Table 3:** Correlation matrix of the identified glucosinolate components under the temperature, humidity, and CO_2_ treatments.

Variables	T_Pro	T_Sin	T_Glu	T_4-met	T_Neo	H_Pro	H_Sin	H_Glu	H_4-met	H_Neo	C_Pro	C_Sin	C_Glu	C_4-met	C_Neo
T_Pro	1.00														
T_Sin	−0.02	1.00						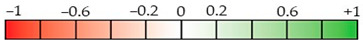							
T_Glu	−0.12	0.96 ***	1.00												
T_4-met	−0.13	−0.65 ***	−0.59 ***	1.00				Strong negative		Not correlated			Strong positive		
T_Neo	0.51***	0.74 ***	0.76 ***	−0.52 ***	1.00										
H_Pro	−0.52 ***	0.24	0.50 ***	−0.08	0.25	1.00									
H_Sin	−0.13	0.08	−0.06	−0.66 ***	−0.26	−0.35 *	1.00								
H_Glu	−0.01	0.53 ***	0.66 ***	0.14	0.65 ***	0.59 ***	−0.79 ***	1.00							
H_4-met	−0.21	−0.54 ***	−0.36 *	0.88 ***	−0.29	0.37 *	−0.82 ***	0.41 **	1.00						
H_Neo	0.00	0.52 ***	0.35 *	0.11	0.16	−0.38 *	−0.13	0.30 *	−0.17	1.00					
C_Pro	0.70 ***	−0.64 ***	−0.69 ***	0.06	−0.11	−0.50 ***	0.13	−0.54 ***	−0.03	−0.53 ***	1.00				
C_Sin	0.37 *	0.68 ***	0.71 ***	−0.84 ***	0.86 ***	0.31 *	0.17	0.30 *	−0.56 ***	−0.17	−0.01	1.00			
C_Glu	0.70 ***	−0.63***	−0.58 ***	0.27	0.04	−0.22	−0.28	−0.16	0.31 *	−0.57 ***	0.90 ***	0.01	1.00		
C_4-met	0.36 *	−0.91 ***	−0.93 ***	0.36 *	−0.52 ***	−0.45 **	0.11	−0.66 ***	0.22	−0.57 ***	0.90 ***	−0.40 **	0.80 ***	1.00	
C_Neo	0.77 ***	−0.50 ***	−0.56 ***	−0.08	0.03	−0.47 **	0.17	−0.49 **	−0.14	−0.53 ***	0.99 ***	0.16	0.88 ***	0.81 ***	1.00
VIF	1.06	4.72	6.46	2.83	1.57	1.32	1.09	1.14	1.47	1.01	1.56	2.39	1.63	2.92	1.28

*, **, *** indicate the 5%, 1%, and 0.1% significance levels, respectively. T_Pro, T_Sin, T_Glu, T_4-met, T_Neo: progoitrin, sinigrin, glucobrassicin, 4-methoxyglucobrassicin, and neoglucobrassicin observed under experiment 1 (temperature effect); H_Pro, H_Sin, H_Glu, H_4-met, H_Neo: progoitrin, sinigrin, glucobrassicin, 4-methoxyglucobrassicin, and neoglucobrassicin observed under experiment 2 (relative humidity effect); C_Pro, C_Sin, C_Glu, C_4-met, C_Neo: progoitrin, sinigrin, glucobrassicin, 4-methoxyglucobrassicin, and neoglucobrassicin observed under experiment 3 (CO_2_ effect), respectively. VIF: variance inflation factor.

**Table 4:** Effects of different temperature levels on kale growth at different cultivation periods.

Sampling Time	Temp. Level (°C)	Growth Variables								
		P_Height (mm)	P_Width (mm)	P_Weight (g)	No_Leaf	Stem dia. (mm)	Chlor_Level (ppm)	L_Length (mm)	L_Width (mm)	L_Weight (g)
2 weeks	14	95.0 ± 4.5 ^a^	161.7 ± 8.1 ^b^	11.9 ± 1.2 ^b^	7.0 ± 0.0 ^a^	2.4 ± 0.1 ^a^	46.9 ± 1.0 ^a^	8.4 ± 0.5 ^c^	7.4 ± 0.4 ^c^	2.9 ± 0.2 ^b^
	17	99.3 ± 4.7 ^a^	186.0 ± 24.3 ^ab^	12.6 ± 0.3 ^a^	6.7 ± 3.7 ^a^	2.5 ± 0.2 ^a^	49.1 ± 0.6 ^a^	10.6 ± 0.2 ^b^	9.4 ± 0.4 ^b^	5.2 ± 0.6 ^a^
	20	84.7 ± 1.2 ^a^	167.0 ± 22.7 ^b^	13.3 ± 0.2 ^a^	7.0 ± 0.1 ^a^	2.7 ± 0.1 ^a^	53.4 ± 5.4 ^a^	13.0 ± 0.4 ^a^	11.3 ± 0.6 ^a^	6.3 ± 0.4 ^a^
	23	92.3 ± 11 ^a^	196.0 ± 14.2 ^ab^	13.0 ± 0.3 ^a^	7.0 ± 0.1 ^a^	2.8 ± 0.9 ^a^	57.0 ± 2.8 ^a^	12.8 ± 0.8 ^a^	11.1 ± 0.6 ^ab^	6.0 ± 1.1 ^a^
	26	90.3 ± 8.9 ^a^	215.0 ± 14.1 ^a^	13.3 ± 0.3 ^a^	7.0 ± 0.8 ^a^	2.8 ± 0.2 ^a^	52.0 ± 0.3 ^a^	12.6 ± 0.9 ^a^	11.3 ± 0.4 ^a^	5.8 ± 0.2 ^a^
4 weeks	14	115.3 ± 8.3 ^b^	274.3 ± 15.0 ^a^	21.3 ± 4.1 ^a^	11.1 ± 0.2 ^a^	13.0 ± 0.0 ^a^	55.5 ± 4.3 ^a^	19.9 ± 0.8 ^c^	12.6 ± 0.46 ^b^	9.2 ± 0.9 ^b^
	17	129.7 ± 5.3 ^b^	260.3 ± 39.4 ^a^	23.6 ± 6.2 ^a^	12.7 ± 0.5 ^a^	14.3 ± 0.5 ^a^	61.2 ± 2.4 ^a^	26.7 ± 0.2 ^b^	17.1 ± 1.1 ^ab^	16.0 ± 3.3 ^ab^
	20	143.3 ± 6.5 ^ab^	286.3 ± 13.5 ^a^	28.3 ± 4.3 ^a^	12.0 ± 0.7 ^a^	15.7 ± 0.4 ^a^	48.4 ± 15.1 ^a^	31.2 ± 2.0 ^ab^	19.0 ± 1.4 ^a^	19.1 ± 6.1 ^a^
	23	137.3 ± 8.6 ^ab^	278.3 ± 36.5 ^a^	25.4 ± 3.2 ^a^	13.5 ± 1.3 ^a^	17.7 ± 0.4 ^a^	58.5 ± 1.7 ^a^	31.8 ± 2.5 ^ab^	20.2 ± 1.8 ^a^	20.0 ± 3.8 ^a^
	26	176.3 ± 27.7 ^a^	277.0 ± 12.8 ^a^	22.2 ± 4.8 ^a^	10.0 ± 0.4 ^a^	15.6 ± 0.9 ^a^	53.6 ± 4.3 ^a^	37.0 ± 2.5 ^a^	19.8 ± 1.5 ^a^	20.1 ± 5.7 ^a^

^a, b, c^ Different letters in the same column indicate a significant difference (*p* ≤ 0.05). P_height: plant height, P_width: plant width, P_weight: plant weight, No_leaf: number of leaves, Stem dia.: stem diameter, Chlor_level: chlorophyll level, L_length: leaf length, L_width: leaf width, L_weight: leaf weight.

**Table 5:** Correlation matrix showing kale growth and glucosinolate variables and their constituents (experiment 1).

Variables	P_Height	P_Width	P_Weight	No_Leaf	Stem dia.	Chlor_Level	L_Length	L_Width	L_Weight	Pro	Sin	Glu	4-Met	Neo
P_height	1													
P_width	0.98 ***	1					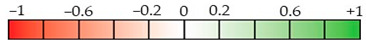							
P_weight	0.81 ***	0.73 ***	1											
No_leaf	0.08	−0.08	0.54 ***	1			Strong negative		Not correlated			Strong positive		
Stem dia.	0.76 ***	0.86 ***	0.36 **	−0.57 ***	1									
Chlor_level	−0.36 **	−0.22	−0.63 ***	−0.65 ***	0.12	1								
L_length	0.85 ***	0.85 ***	0.78 ***	−0.04	0.76 ***	−0.50 ***	1							
L_width	0.60 ***	0.60 ***	0.63 ***	−0.1	0.63 ***	−0.51 ***	0.93 ***	1						
L_weight	0.55 ***	0.52 ***	0.66 ***	0.05	0.49 ***	−0.64 ***	0.90 ***	0.98 ***	1					
Pro	−0.60 ***	−0.55 ***	−0.66 ***	−0.13	−0.45 ***	0.78 ***	−0.88 ***	−0.93 ***	−0.96 ***	1				
Sin	−0.68 ***	−0.70 ***	−0.38 **	0.32 *	−0.76 ***	0.43 ***	−0.83 ***	−0.77 ***	−0.72 ***	0.81 ***	1			
Glu	−0.96 ***	−0.96 ***	−0.76 ***	0.06	−0.84 ***	0.41 **	−0.95 ***	−0.77 ***	−0.72 ***	0.75 ***	0.83 ***	1		
4-Met	0.52 ***	0.52 ***	0.38 **	−0.20	0.57 ***	−0.59 ***	0.81 ***	0.86 ***	0.85 ***	−0.92 ***	−0.94 ***	−0.72 ***	1	
Neo	−0.21	−0.33 *	0.33 *	0.68 ***	−0.54 ***	−0.74 ***	0.10	0.31 *	0.47 ***	−0.46 ***	0.10	0.14	0.20	1
VIF	3.47	4.08	3.65	1.06	4.21	2.16	16.67	4.64	2.99	3.09	5.81	11.99	3.46	1.01

*, **, *** indicate the 5%, 1%, and 0.1% significance levels, respectively. P_height: plant height, P_width: plant width, P_weight: plant weight, No_leaf: number of leaves, Stem dia.: stem diameter, Chlor_level: chlorophyll level, L_length: leaf length, L_width: leaf width, L_weight: leaf weight, Pro: progoitrin, Sin: sinigrin, Glu: glucobrassicin, 4-Met: 4-methoxyglucobrassicin, and Neo: neoglucobrassicin. VIF: variance inflation factor.

**Table 6:** Effects of different relative humidity levels on kale growth in different cultivation periods.

Sampling Time	Humi. Level (%)	Growth Variables								
		P_Height (mm)	P_Width (mm)	P_Weight (g)	No_Leaf	Stem Dia. (mm)	Chlor_Level (ppm)	L_Length (mm)	L_Width (mm)	L_Weight (g)
2 weeks	45	68.7 ± 7.6 ^b^	171.7 ± 8.1 ^b^	5.5 ± 0.1 ^c^	8.0 ± 0.0 ^ab^	2.9 ± 0.2 ^c^	60.5 ± 4.3 ^a^	97.7 ± 9.5 ^c^	58.0 ± 2.0 ^c^	1.1 ± 0.1 ^c^
	55	74.3 ± 10.3 ^b^	196.0 ± 24.3 ^ab^	8.2 ± 1.2 ^ab^	8.7 ± 0.6 ^a^	3.8 ± 0.3 ^a^	66.2 ± 2.4 ^a^	111.7 ± 9.5 ^bc^	67.7 ± 5.5 ^bc^	1.8 ± 0.4 ^b^
	65	79.3 ± 5.1 ^ab^	177.0 ± 22.6 ^b^	6.3 ± 1.4 ^bc^	7.7 ± 0.6 ^b^	3.3 ± 0.2 ^b^	53.4 ± 15.1 ^a^	114.0 ± 5.6 ^b^	67.3 ± 5.1 ^bc^	1.8 ± 0.2 ^b^
	75	83.0 ± 1.0 ^ab^	206.0 ± 14.2 ^ab^	7.7 ± 1.2 ^abc^	8.0 ± 0.1 ^ab^	3.2 ± 0.2 ^bc^	63.5 ± 1.7 ^a^	133.3 ± 9.1 ^a^	76.7 ± 4.6 ^ab^	2.1 ± 0.3 ^ab^
	85	93.3 ± 6.8 ^a^	225.0 ± 14.1 ^a^	8.9 ± 1.5 ^a^	8.0 ± 0.0 ^ab^	3.5 ± 0.1 ^ab^	58.6 ± 4.3 ^a^	132.3 ± 8.6 ^a^	81.7 ± 8.4 ^a^	2.5 ± 0.3 ^a^
4 weeks	45	114.3 ± 9.3 ^b^	291.7 ± 62.8 ^b^	23.4 ± 10.2 ^a^	13.0 ± 1.0 ^bc^	5.0 ± 0.7 ^c^	66.1 ± 1.6 ^a^	158.7 ± 25.2 ^b^	105.3 ± 21.7 ^a^	5.3 ± 1.3 ^b^
	55	119.7 ± 15.3 ^b^	302.0 ± 18.1 ^b^	29.4 ± 3.5 ^a^	13.3 ± 0.6 ^ab^	5.2 ± 0.3 ^bc^	65.3 ± 1.8 ^a^	171.0 ± 14.1 ^ab^	118.0 ± 7.9 ^a^	8.9 ± 1.5 ^a^
	65	133.3 ± 16.5 ^ab^	314.0 ± 18.0 ^b^	33.0 ± 4.9 ^a^	14.7 ± 0.6 ^a^	5.0 ± 0.2 ^c^	47.1 ± 2.8 ^a^	172.3 ± 17.1 ^ab^	111.3 ± 9.1 ^a^	9.1 ± 1.1 ^a^
	75	125.3 ± 9.6 ^b^	316.3 ± 7.6 ^b^	25.7 ± 0.9 ^a^	11.7 ± 0.6 ^c^	5.7 ± 0.3 ^ab^	61.3 ± 3.1 ^a^	186.3 ± 6.4 ^ab^	114.7 ± 9.7 ^a^	8.9 ± 2.2 ^a^
	85	166.3 ± 37.9 ^a^	383.3 ± 10.1 ^a^	34.7 ± 6.9 ^a^	13.0 ± 1.0 ^bc^	6.1 ± 0.1 ^a^	59.2 ± 3.1 ^a^	191.0 ± 11.5 ^a^	126.0 ± 12.5 ^a^	8.7 ± 2.3 ^a^

^a, b, c^ Different letters in the same column indicate significant differences (*p* ≤ 0.05). P_height: plant height, P_width: plant width, P_weight: plant weight, No_leaf: number of leaves, Stem dia.: stem diameter, Chlor_level: chlorophyll level, L_length: leaf length, L_width: leaf width, L_weight: leaf weight.

**Table 7:** Correlation matrix showing kale growth and glucosinolate variables and their constituents (experiment 2).

Variables	P_Height	P_Width	P_Weight	No_Leaf	Stem Dia.	Chlor_Level	L_Length	L_Width	L_Weight	Pro	Sin	Glu	4-Met	Neo
P_height	1													
P_width	0.99 ***	1					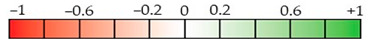							
P_weight	0.81 ***	0.73 ***	1											
No_leaf	0.08	−0.08	0.54 ***	1			Strong negative		Not correlated			Strong positive		
Stem dia.	0.76 ***	0.86 ***	0.36 **	−0.57 ***	1									
Chlor_level	−0.36 **	−0.22	−0.63 ***	−0.65 ***	0.12	1								
L_length	0.76 ***	0.81 ***	0.53 ***	−0.39 **	0.91 ***	−0.21	1							
L_width	0.80 ***	0.85 ***	0.71 ***	−0.14	0.81 ***	−0.02	0.82 ***	1						
L_weight	0.42 ***	0.40 **	0.67 ***	0.12	0.37 **	−0.50 ***	0.69 ***	0.65 ***	1					
Pro	−0.17	−0.13	−0.60 ***	−0.50 ***	0.09	0.09	−0.04	−0.51 ***	−0.54 ***	1				
Sin	0.61 ***	0.62 ***	0.38 **	−0.29 *	0.69 ***	−0.50 ***	0.85 ***	0.42 ***	0.55 ***	0.34 **	1			
Glu	−0.49 ***	−0.59 ***	0.04	0.74 ***	−0.85 ***	−0.13	−0.72 ***	−0.37 **	−0.05	−0.60 ***	−0.76 ***	1		
4-Met	−0.39 **	−0.35 **	−0.47 ***	−0.03	−0.40 **	0.73 ***	−0.68 ***	−0.31 *	−0.79 ***	−0.05	−0.86 ***	0.35 **	1	
Neo	0.28 *	0.20	0.70 ***	0.46 ***	0.03	−0.68 ***	0.41 **	0.42 ***	0.93 ***	−0.64 ***	0.34 **	0.28*	−0.72 ***	1
VIF	3.47	4.07	2.65	1.06	4.21	1.16	11.93	2.56	1.78	1.11	4.97	2.01	1.42	1.22

*, **, *** indicates 5%, 1%, and 0.1% significance levels, respectively. P_height: plant height, P_width: plant width, P_weight: plant weight, No_leaf: number of leaves, Stem dia.: stem diameter, Chlor_level: chlorophyll level, L_length: leaf length, L_width: leaf width, L_weight: leaf weight, Pro: progoitrin, Sin: sinigrin, Glu: glucobrassicin, 4-met: 4-methoxyglucobrassicin, and Neo: neoglucobrassicin. VIF: variance inflation factor.

**Table 8:** Effects of different carbon dioxide levels on kale growth at different cultivation periods.

Sampling Time	CO_2_ Level (ppm)	Growth Variables								
		P_Height (mm)	P_Width (mm)	P_Weight (g)	No_Leaf	Stem Dia. (mm)	Chlor_Level (ppm)	L_Length (mm)	L_Width (mm)	L_Weight (g)
2 weeks	400	85.0 ± 4.5 ^a^	213.0 ± 9.2 ^a^	11.0 ± 1.8 ^a^	4.2 ± 0.3 ^a^	11.3 ± 0.4 ^a^	142.0 ± 10.0 ^a^	53.0 ± 4.2 ^a^	60.1 ± 1.4 ^a^	3.5 ± 0.2 ^a^
	700	89.3 ± 4.7 ^a^	228.0 ± 11.7 ^a^	11.3 ± 1.7 ^a^	4.2 ± 0.2 ^a^	11.7 ± 0.4 ^a^	140.7 ± 5.4 ^a^	47.7 ± 1.2 ^a^	63.0 ± 4.3 ^a^	3.6 ± 1.1 ^a^
	1000	74.7 ± 1.2 ^a^	196.3 ± 16.8 ^a^	10.4 ± 1.4 ^a^	4.3 ± 0.3 ^a^	11.7 ± 0.4 ^a^	127.0 ± 6.1 ^a^	50.0 ± 4.0 ^a^	64.1 ± 3.2 ^a^	3.4 ± 0.4 ^a^
	1300	82.3 ± 11.0 ^a^	211.3 ± 4.5 ^a^	8.9 ± 0.4 ^a^	4.0 ± 0.1 ^a^	11.0 ± 0.8 ^a^	125.0 ± 1.4 ^a^	45.3 ± 4.1 ^a^	69.8 ± 1.1 ^a^	3.2 ± 0.6 ^a^
	1600	80.3 ± 8.9 ^a^	194.0 ± 17.2 ^a^	7.5 ± 0.6 ^a^	4.0 ± 0.3 ^a^	11.7 ± 0.9 ^a^	123.0 ± 7.2 ^a^	52.3 ± 3.2 ^a^	66.8 ± 1.3 ^a^	2.9 ± 0.2 ^a^
4 weeks	400	111.7 ± 6.5 ^a^	284.3 ± 13.0 ^a^	22.2 ± 4.8 ^a^	7.0 ± 0.4 ^a^	14.7 ± 0.9 ^a^	186.0 ± 14.3 ^a^	68.0 ± 2.1 ^a^	62.7 ± 2.8 ^a^	5.7 ± 1.7 ^a^
	700	105.3 ± 4.7 ^ab^	270.3 ± 49.4 ^a^	28.4 ± 3.2 ^a^	7.1 ± 0.3 ^a^	15.7 ± 0.4 ^a^	179.0 ± 9.9 ^a^	67.3 ± 4.7 ^a^	66.9 ± 1.3 ^a^	5.3 ± 0.8 ^a^
	1000	102.7 ± 4.9 ^ab^	296.3 ± 6.5 ^a^	25.0 ± 4.3 ^a^	7.4 ± 0.4 ^a^	15.7 ± 0.4 ^a^	175.7 ± 5.2 ^a^	70.3 ± 2.4 ^a^	65.6 ± 0.8 ^a^	4.5 ± 0.1 ^a^
	1300	99.3 ± 5.5 ^ab^	288.3 ± 36.5 ^a^	23.6 ± 6.2 ^a^	6.7 ± 0.5 ^a^	14.3 ± 0.5 ^a^	169.3 ± 12.0 ^a^	63.0 ± 2.0 ^a^	67.7 ± 1.2 ^a^	4.7 ± 1.3 ^a^
	1600	93.0 ± 5.0 ^b^	288.0 ± 12.8 ^a^	25.3 ± 4.1 ^a^	7.1 ± 0.2 ^a^	15.0 ± 0.0 ^a^	179.0 ± 11.3 ^a^	68.3 ± 4.1 ^a^	66.4 ± 2.4 ^a^	4.9 ± 0.9 ^a^

^a, b^ Different letters in the same column indicate significant differences (*p* ≤ 0.05). P_height: plant height, P_width: plant width, P_weight: plant weight, No_leaf: number of leaves, Stem dia.: stem diameter, Chlor_level: chlorophyll level, L_length: leaf length, L_width: leaf width, L_weight: leaf weight.

**Table 9:** Correlation matrix showing kale growth and glucosinolate variables and their constituents (experiment 3).

Variables	P_Height	P_Width	P_Weight	No_Leaf	Stem Dia.	Chlor_Level	L_Length	L_Width	L_Weight	Pro	Sin	Glu	4-Met	Neo
P_height	1													
P_width	−0.32 *	1					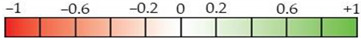							
P_weight	−0.16	−0.87 ***	1											
No_leaf	0.06	0.20	−0.37 **	1			Strong negative		Not correlated			Strong positive		
Stem dia.	0.09	−0.19	0.01	0.88 ***	1									
Chlor_level	0.56 ***	−0.31 *	0.08	0.29 *	0.16	1								
L_length	0.15	0.21	−0.38 **	0.94 ***	0.72 ***	0.57 ***	1							
L_width	−0.69 ***	−0.12	0.41 **	−0.22	0.08	−0.84 ***	−0.50 ***	1						
L_weight	−0.16	−0.87 ***	1.00 ***	−0.37 **	0.01	0.08	−0.38 **	0.41 **	1					
Pro	−0.58 ***	0.25	0.10	−0.75 ***	−0.70 ***	−0.78 ***	−0.84 ***	0.63 ***	0.10	1				
Sin	0.92 ***	−0.04	−0.43 ***	0.01	−0.03	0.25	0.03	−0.56 ***	−0.43 ***	−0.36 **	1			
Glu	−0.50 ***	0.19	0.19	−0.80 ***	−0.88 ***	−0.27 *	−0.66 ***	0.2	0.19	0.80 ***	−0.45 ***	1		
4-Met	−0.65 ***	−0.05	0.48 ***	−0.52 ***	−0.55 ***	−0.03	−0.37 **	0.23	0.48 ***	0.53 ***	−0.76 ***	0.85 ***	1	
Neo	−0.54 ***	−0.24	0.64 ***	−0.66 ***	−0.59 ***	−0.05	−0.53 ***	0.29 *	0.64 ***	0.56 ***	−0.68 ***	0.85 ***	0.97 ***	1
VIF	4.23	1.22	1.51	1.75	1.03	1.62	1.79	1.5	1.51	1.82	3.81	6.85	3.15	7.9

*, **, *** indicates the 5%, 1%, and 0.1% significance level, respectively. P_height: plant height, P_width: plant width, P_weight: plant weight, No_leaf: number of leaves, Stem dia.: stem diameter, Chlor_level: chlorophyll level, L_length: leaf length, L_width: leaf width, L_weight: leaf weight, Pro: progoitrin, Sin: sinigrin, Glu: glucobrassicin, 4-Met: 4-methoxyglucobrassicin, and Neo: neoglucobrassicin. VIF: variance inflation factor.

## Data Availability

All the data reported here are available from the authors upon request.
